# The role of the adipocytokines vaspin and visfatin in vascular endothelial function and insulin resistance in obese children

**DOI:** 10.1186/s12902-019-0452-6

**Published:** 2019-11-26

**Authors:** Chunyan Yin, Wei Hu, Ming Wang, Yanfeng Xiao

**Affiliations:** Department of Pediatrics, The Second Affiliated Hospital of Xi’an Jiaotong University, No. 157 of West 5th Road, Xi’an, ShanXi 710049 People’s Republic of China

**Keywords:** Obese children, Vaspin, Visfatin, Endothelial dysfunction, Vascular inflammation

## Abstract

**Background:**

We measured the concentrations of the adipocytokines vaspin and visfatin in obese Chinese children. Furthermore, we studied the correlation of these adipocytokines with early-onset metabolic and vascular sequelae among these children.

**Methods:**

A total of 244 children (160 obese and 84 lean) were included in this study. Vaspin and visfatin were detected using enzyme-linked immunosorbent assays. We also assayed other metabolic and cardiovascular parameters. The associations of serum vaspin and visfatin concentrations with metabolic and cardiovascular parameters were determined.

**Results:**

We found a significant elevation in the concentrations of vaspin and visfatin in obese children compared to the concentrations in lean children. Additionally, we found a significant positive correlation between visfatin and vaspin levels, as well as inflammatory cell infiltration and markers of endothelial activation, but these factors did not affect insulin resistance in obese children. Multiple regression analyses confirmed that vaspin is the strongest predictor of higher tumour necrosis factor-α (TNF-α), interleukin-6 (IL-6), angiotensin-2 (Ang-2), vascular cellular adhesion molecule-1 (VCAM-1), and E-selectin levels. We also found a significant association between visfatin and Ang-2, IL-6, VCAM-1, and E-selectin levels.

**Conclusion:**

The adipocytokines vaspin and visfatin are significantly interrelated, and both adipocytokines play a role in vascular endothelial function and inflammation.

## Background

Obesity is a growing health concern affecting more than 711.4 million individuals globally [1], among whom 107.7 million are children. Obesity is one of the major risk factors for a variety of chronic diseases, and its prevalence has been shown to increase with age in children [2]. Obesity leads to hypertension, insulin resistance, and diabetes [3, 4]. With the increasing prevalence of childhood obesity, these comorbidities have begun to develop in early childhood.

Due to a high body mass index (BMI), cardiovascular disease (CVD) alone accounted for 2.7 million deaths and 66.3 million disabilities [1]. The primary signs of CVD are endothelial dysfunction and subclinical inflammation in adults [5]. However, various biomarkers, including tumour necrosis factor-α (TNF-α), C-reactive protein (CRP), and cellular adhesion molecules (CAMs), have been reported to be significantly elevated in the plasma of obese children [6, 7]. This elevation indicates the presence of vascular inflammation and endothelial dysfunction in childhood. Previous studies have shown that the occurrence of endothelial dysfunction and subclinical inflammation in childhood is considered to be the first sign of CVD development in adulthood [8].

Several adipocytokines have also been identified in the context of obesity [9]. Hence, understanding the diverse effects of distinct adipocytokines, as well as the relationships between these bioactive mediators, will help elucidate the underlying molecular basis of obesity-related diseases. Previous studies have shown that vaspin and visfatin are two adipocytokines that are closely associated with insulin resistance [10]. Vaspin belongs to a family of serine protease inhibitors (serpins), and it impairs glucose tolerance, leading to insulin resistance in obese mice [11]. In addition, elevated vaspin serum concentrations have been associated with obesity and impaired insulin sensitivity in adults [12]. Previous research reported that visfatin induces TNF-α and interleukin (IL)-6 in human monocytes [13]. It has also been demonstrated that recombinant visfatin directly binds to the insulin receptor (IR), leading to tyrosine phosphorylation and resulting in enhanced glucose uptake [14]. However, research conducted to understand the role of these adipocytokines in the pathogenesis of insulin resistance in the context of childhood obesity is limited. There has been no investigation to date to understand the association of vaspin and visfatin with endothelial dysfunction and vascular inflammation in obese children. Children either present earlier stages of pathogenesis or are relatively free of interfering comorbidities. In this regard, it is important to conduct studies in children to gain better insight into the association of vaspin and visfatin with the early stages of obesity-related disease.

In this study, we compared vaspin and visfatin levels between obese children and healthy controls. We also investigated the correlation of these two adipocytokines with one another. Finally, we investigated the association of these adipocytokines with metabolic syndrome, as this is a known cardiovascular risk factor and maker of endothelial activation in Chinese children.

## Methods

### Subjects

A total of 244 Chinese children, including 160 obese and 84 lean children, were included in this study. Children visiting the obesity clinic of the Pediatric Department of the Second Affiliated Hospital of Xi’an Jiao Tong University and the medical examination centre for routine check-ups were recruited as the study group and control group, respectively. Children with a BMI >90th percentile and < 85th percentile for their age and sex were included in the obese and lean groups, respectively [15]. Children with endocrine disorders, genetic obesity syndromes, acute and chronic infectious disease, or autoimmune diseases were excluded from this study. We obtained approval for this study from the institutional ethics committee. The study was conducted in accordance with the Declaration of Helsinki and was approved by the ethics committee of the Second Affiliated Hospital of Xi’an JiaoTong University. All parents and children were carefully informed of the study protocol and provided written informed consent, which was duly signed before the subjects participated in the study.

### Anthropometric measurements

Standing height and body weight were measured with children wearing light clothing without shoes. Waist and hip circumferences (WC and HC, respectively) were measured to the nearest 0.1 cm. The oscillometric device OMRON705IT was used to measure systolic and diastolic blood pressures (SBP and DBP, respectively) [16]. The Tanner criteria were used to determine pubertal developmental stages. Children were divided into two groups based on the criteria: prepubertal (Tanner stage I) and pubertal (Tanner stage II–V) [17]. None of the participants were taking any form of medication. We defined insulin resistance as HOMA-IR > 2 [18].

A body shape index (ABSI) was calculated using the following formula:

ABSI = WC (m)/ [BMI^2/3^(kg/m^2^) × Height^1/2^ (m)] [19].

The visceral adiposity index (VAI) was calculated using the following formula:

VAI_male_ = [WC (cm)/39.68–1.88BMI(kg/m^2^)][TGs (mmol/L)/1.03, 1.31/HDL (mmol/L)].

VAI_female_ = [WC (cm)/36.58–1.89BMI(kg/m^2^)][TGs (mmol/L)/0.81, 1.52/HDL (mmol/L)].

TGs: triglycerides; HDL: high-density lipoprotein.

### Blood sampling and analysis

Blood samples were taken in the morning at 8:00 AM after 12 h of overnight fasting. Blood specimens were centrifuged, and the remaining serum was stored at − 80 °C. The oral glucose tolerance test (OGTT) was performed with 1.75 g glucose per kg of body weight (maximum of 75 g glucose) after a 12 h overnight fast. Blood specimens were obtained at 0 and 120 min to measure glucose and insulin concentrations. A double-antibody radioimmunoassay (RIA) was used to measure plasma insulin. An automatic analyser was used to determine the plasma glucose and lipid profiles (total cholesterol (TC), TGs, low-density lipoprotein (LDL), and HDL).

Serum IL-6 and TNF-α concentrations were measured using a chemiluminescent enzyme assay (Fujirebio Inc., Tokyo, Japan). CRP was determined using an enzymatic latex-enhanced immunonephelometric assay (Dade Behring, Tokyo, Japan). Plasma levels of soluble VCAM-1, soluble ICAM, E-selectin, angiotensin-2 (Ang-2), adiponectin, and obestatin were detected using enzyme-linked immunosorbent assays (ELISAs) (USCN Life Science Inc., Wuhan, China). Vaspin and visfatin concentrations were also measured using ELISAs (Excell, Shanghai, China). The vaspin and visfatin inter- and intra-assay CVs were 4.8 and 7.2% and 5.6 and 8.3%, respectively.

### Statistical analysis

The normally distributed data are presented as the means ± SEMs. Non-parametrically distributed parameters were log-transformed before analysis. Differences between two groups were assessed using Student’s two-tailed t-test or the χ^2^ test. General linear modelling was used to analyse the correlation between visfatin and vaspin with other biochemical parameters. Sex and age were controlled as confounding factors. For multiple regression analyses, anthropometric indicators and adipocytokines, as well as inflammatory factors including high-sensitivity CRP (hs-CRP), IL-6, and TNF-α, and adhesion molecules including ICAM-1 and E-selectin, were used as the independent variables and dependent variables, respectively. Statistical analyses were performed using the Statistical Package for the Social Science (SPSS) 20.0 software (SPSS Inc., Chicago, IL, USA). *P* < 0.05 was considered statistically significant.

## Results

### Subject characteristics

Table 1 shows the clinical characteristics of the study subjects. A total of 160 obese and 84 lean children were included in this study. As expected, there were no significant differences in age, height, or sex between the two groups. BMI, SDS-BMI, waist:hip ratio, WC, VAI, ABSI, WHtR, SBP, DBP, SDS-SBP, SDS-DBP, insulin, 2-h insulin, HOMA-IR, TGs, and LDL cholesterol were significantly elevated in the obese children. FPG, 2-h PG, TC, and HDL-cholesterol were not significantly different between the two groups, but plasma levels of adiponectin and obestatin were significantly lower in obese children than in lean children. We found significant elevations in plasma levels of VCAM-1, ICAM-1, E-selectin, and Ang-2 in obese children compared to the levels in lean children. In addition, obese children had higher plasma levels of IL-6, hs-CRP, and TNF-α than lean children. Serum visfatin and vaspin levels were significantly elevated in obese children compared to the levels in lean children (Table 1).

**Table 1:** Clinical and laboratory characteristics of the study population

Characteristic	Lean (*N* = 80)	Obese (*N* = 160)
Age (y)	10.31 ± 2.45	11.03 ± 2.76
BMI (kg/m^2^)	17.35 ± 2.05	28.26 ± 3.84^b^
SDS-BMI	0.42 ± 0.78	2.85 ± 0.92^b^
Weight (kg)	38.65 ± 7.26	60.03 ± 12.26^b^
Height (m)	1.48 ± 0.32	1.52 ± 0.28
WHtR	0.42 ± 0.21	0.65 ± 0.26^b^
WC (cm)	63.45 ± 7.06	93.56 ± 11.28^b^
WHR	0.79 ± 0.06	0.96 ± 0.07^b^
SBP (mm Hg)	92.2 ± 5.6	108.6 ± 12.7^b^
DBP (mm Hg)	76.5 ± 3.6	80.8 ± 4.5^a^
SDS-SBP	1.49 ± 0.83	1.91 ± 0.92^b^
SDS-DBP	0.93 ± 0.41	1.22 ± 0.73^b^
FPG (mmol/L)	4.36 ± 0.82	5.13 ± 0.63
2-h PG (mmol/L)	6.08 ± 1.03	6.83 ± 1.27
Insulin (lU/mL)	12.0 ± 2.9	15.8 ± 4.9 ^a^
2-h Insulin (lU/mL)	73.5 ± 8.5	79.1 ± 11.2 ^a^
HOMA-IR	2.63 (1.56, 4.38)	3.29 (2.21, 5.69)^a^
TC (mmol/L)	3.94 ± 0.63	3.89 ± 0.55
TGs (mmol/L)	1.05 ± 0.31	1.43 ± 0.62^a^
HDL-C (mmol/L)	1.41 ± 0.46	1.39 ± 0.35
LDL-C (mmol/L)	2.31 ± 0.93	2.46 ± 1.01^a^
Adiponectin (μg/mL)	11.28 ± 1.53	6.38 ± 0.96^b^
Obestatin (pg/mL)	236.32 ± 30.54	135.04 ± 24.36^b^
Vaspin (μg/mL)	4.87 ± 0.93	10.81 ± 1.07^b^
Visfatin (μg/mL)	36.54 ± 9.31	73.60 ± 14.86^b^
hsCRP (ng/mL)	1086.58 ± 165.61	1423.05 ± 221.36^b^
IL-6 (pg/mL)	18.34 ± 4.29	32.42 ± 6.71^b^
TNF-α (ng/mL)	25.16 ± 5.34	50.24 ± 13.43^b^
ICAM-1 (μg/mL)	6.21 ± 1.14	11.36 ± 1.56^b^
VCAM-1 (μg/mL)	153.49 ± 22.53	251.34 ± 38.57^b^
Ang-2 (pg/mL)	83.56 ± 10.28	121.38 ± 16.58^b^
E-selectin (ng/mL)	15.36 ± 3.59	32.53 ± 9.05^b^

Abbreviations: *BMI*, Body mass index; SDS-BMI, BMI s.d. score; *WHtR*, Waist-height ratio; *VAI*, Visceral adiposity index; *ABSI*, A body shape index; *WC*, waist circumference; *WHR*, Waist-to-hip ratio; *SBP*, Systolic blood pressure; *DBP*, Diastolic blood pressure; SDS-SBP, SBP s.d. score; SDS-DBP, DBP s.d. score; *FPG*, Fasting plasma glucose; *HDL-C*, High-density lipoprotein cholesterol; *HOMA-IR*, Homoeostasis model of insulin resistance; *LDL-C*, Low-density lipoprotein; *TC*, Total cholesterol; *TGs*, Triglycerides; *TNF-α*, Tumour necrosis factor-α; *hsCRP*, High-sensitivity C-reactive protein; *ICAM-1*, Intercellular adhesion molecule-1; *IMT*, Intima-media thickness; *VCAM-1*, Vascular cell adhesion molecule-1; Ang-2, angiotensin-2

Data are expressed as the mean ± s.d. or median (25th percentile, 75th percentile). ^a^*P* < 0.05; ^b^*P* < 0.01 compared with obese children

There were no significant differences between male and female children in any of the above indicators, including BMI, SDS-BMI, SDS-SBP, SDS-DBP, insulin, 2-h insulin, HOMA-IR, visfatin, vaspin VCAM-1, ICAM-1, E-selectin, and Ang-2 (Additional file 1: Table S1). There were no significant differences in visfatin, vaspin VCAM-1, ICAM-1, E-selectin, and Ang-2 among participants in different Tanner stages (Additional file 2: Table S2).

We divided the obese children into an insulin resistance group and a non-insulin resistance group according to insulin HOMA-IR z-scores to evaluate the potential correlations of visfatin and vaspin with insulin resistance. We also compared differences in the above indicators between the two groups. We found that plasma levels of visfatin, vaspin, VCAM-1, ICAM-1, E-selectin, and Ang-2 were similar in the insulin resistance and non-insulin resistance groups (Additional file 3: Table S3).

### Relationship of serum vaspin and visfatin levels with other parameters

Table 2 shows the correlations of serum visfatin and vaspin with other indicators in obese children. Significant positive correlations were detected between vaspin and SDS-BMI, BMI, hsCRP, IL-6, TNF-α, VCAM-1, ICAM-1, Ang-2, and E-selectin levels in the single linear correlation analysis. A similar difference was also found for visfatin.

**Table 2:** Simple linear regression analysis using vaspin and visfatin as independent variables in obese children

Variable	Vaspin (μg/mL)		Visfatin (μg/mL)	
	*β*(r)	*P*	*β*(r)	*P*
Anthropometric Parameters				
BMI (kg/m^2^)	0.468	0.012	0.523	0.008
SDS-BMI	0.519	0.009	0.601	0.005
WC (cm)	0.244	0.045	0.258	0.041
WHR	0.122	0.094	0.182	0.075
WHtR	−0.045	0.703	0.055	0.644
VAI	− 0.019	0.872	0.002	0.985
ABSI	0.061	0.606	0.032	0.784
Metabolic parameters				
FPG (mmol/L)	−0.135	0.259	− 0.145	0.224
2 h FPG (mmol/L)	0.147	0.089	0.106	0.133
Insulin (pmol/L)	−0.196	0.085	−0.218	0.072
2-h Insulin (lU/mL)	0.224	0.064	0.187	0,073
Ln HOMA-IR	−0.217	0.067	−0.223	0.060
LDL-C (mmol/L)	−0.205	0.052	−0.019	0.860
HDL-C (mmol/L)	0.032	0.966	0.046	0.721
Ln TGs (mmol/L)	0.021	0.862	0.033	0.784
TC (mmol/L)	0.201	0.090	0.188	0.113
Endothelial and inflammatory markers				
SBP (mm Hg)	0.002	0.987	0.004	0.970
DBP (mm Hg)	0.023	0.848	0.073	0.573
SDS-SBP	0.052	0.654	0.038	0.753
SDS-DBP	−0.053	0.653	0.062	0.603
Ang-2 (pg/mL)	0.914	0.000	0.933	0.000
Adiponectin (μg/mL)	0.019	0.857	−0.088	0.456
Obestatin (pg/mL)	0.060	0.612	0.034	0.766
TNF-α (pg/mL)	0.724	0.000	0.733	0.000
hsCRP (ng/mL)	0.621	0.000	0.633	0.000
IL-6 (pg/mL)	0.739	0.000	0.736	0.000
ICAM-1 (μg/mL)	0.933	0.000	0.953	0.000
E-selectin (ng/mL)	0.843	0.000	0.896	0.000
VCAM-1 (μg/mL)	0.772	0.000	0.726	0.000

### Multiple regression analysis to determine associations of vaspin and vastatin in the Chinese obese cohort

Using multiple regression analyses, we found that vaspin was an independent predictor of hsCRP and IL-6, explaining 8 and 4% of hsCRP and IL-6 variability, respectively. We also found that SDS-BMI was the strongest predictor of hsCRP and IL-6, contributing 29 and 20% to the variability, respectively. Moreover, we confirmed visfatin as an independent predictor of TNF-α and IL-6 in addition to SDS-BMI and WC. Finally, we confirmed that both vaspin and visfatin were the strongest predictors of ICAM-1, E-selectin, and Ang-2 levels (Tables 3 and 4).

**Table 3:** Multiple regression analyses of independent associations of vaspin and visfatin levels with inflammatory parameters in the Chinese Obese Cohort (*N* = 160)

Variables				
Dependent	Independent	Parameter	*β*	P
TNF-α	SDS- BMI, WC, vaspin, visfatin hsCRP, IL-6	SDS- BMI	0.523	0.000
		WC	0.285	0.002
		vaspin	0.367	0.000
		visfatin	0.132	0.058
		IL-6	0.087	0.472
		hsCRP	0.066	0.574
hsCRP	SDS-BMI,WC, vaspin,visfatin, TNF-α, IL-6	SDS- BMI	0.582	0.000
		WC	0.228	0.003
		vaspin	0.205	0.006
		visfatin	0.105	0.085
		TNF-α	0.079	0.568
		IL-6	0.045	0.637
IL-6	SDS-BMI,WC, vaspin,visfatin, TNF-α, hsCRP	SDS- BMI	0.638	0.000
		WC	0.241	0.002
		vaspin	0.184	0.038
		visfatin	0.427	0.000
		TNF-α	0.079	0.532
		hsCRP	0.034	0.876

**Table 4:** Multiple regression analyses of independent associations of vaspin and visfatin levels with endothelial parameters in the Chinese Obese Cohort (N = 160)

Variables				
Dependent	Independent	Parameter	*β*	P
ICAM-1	SDS- BMI, WC, vaspin, visfatin, E-selectinAng-2	SDS- BMI	0.435	0.000
		WC	0.187	0.037
		vaspin	0.326	0.000
		visfatin	0.288	0.002
		E-selectin	0.075	0.514
		Ang-2	0.053	0.731
E-selectin	SDS-BMI,WC, vaspin,visfatin, ICAM-1, Ang-2	SDS- BMI	0.369	0.000
		WC	0.215	0.004
		vaspin	0.174	0.042
		visfatin	0.228	0.003
		ICAM-1	0.067	0.612
		Ang-2	0.032	0.893
Ang-2	SDS-BMI,WC, vaspin,visfatin, ICAM-1, E-selectin	SDS- BMI	0.337	0.000
		WC	0.274	0.003
		vaspin	0.185	0.032
		visfatin	0.164	0.043
		ICAM-1	0.058	0.729
		E-selectin	0.029	0.916

### The association between visfatin and vaspin levels in obese children

**Fig. 1:**
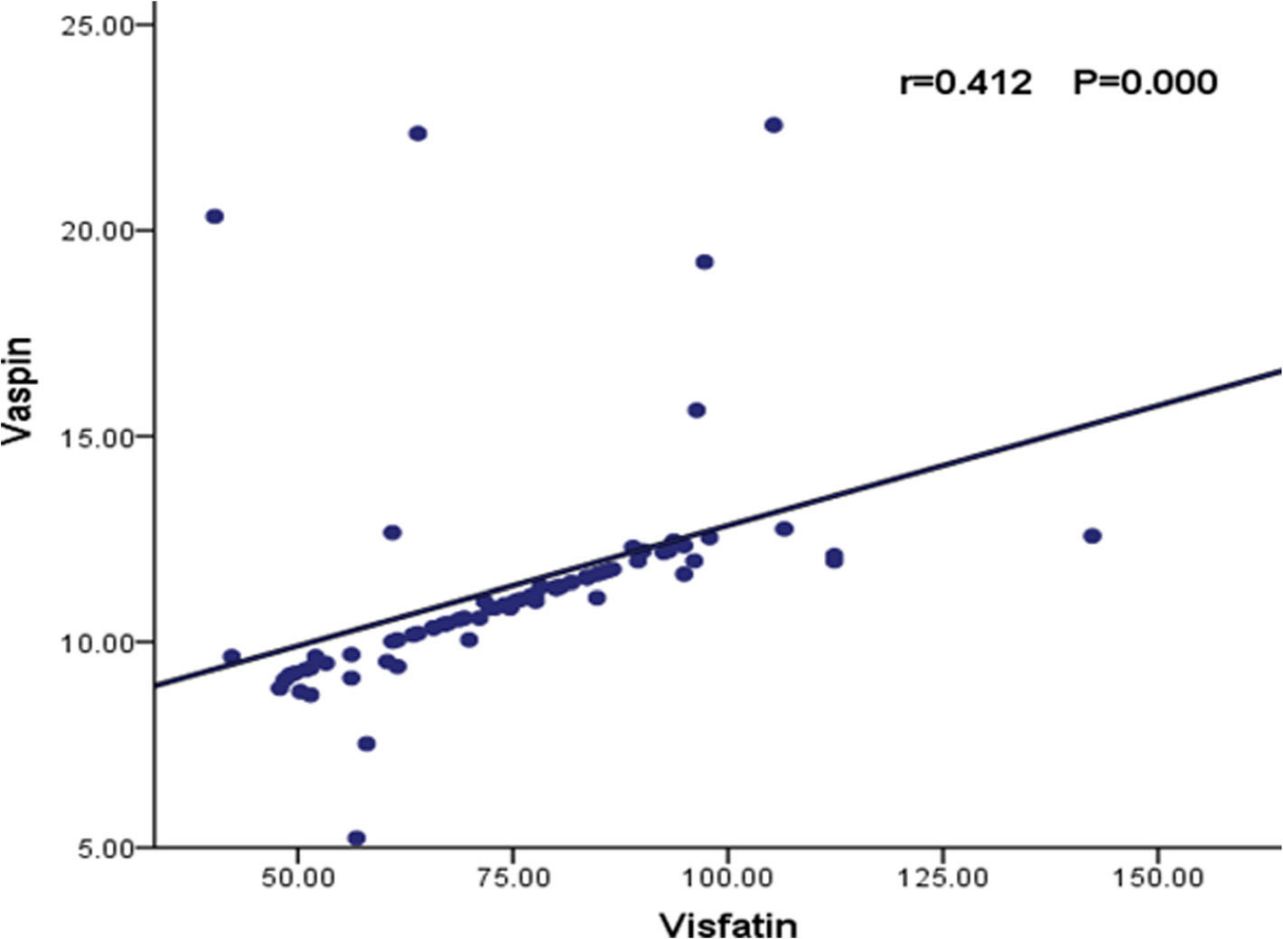
Associations between visfatin and vaspin levels in obese children. Data are expressed as mean ± s.d

We determined the associations between serum visfatin and vaspin levels using simple regression analysis. We found a significant positive correlation between the serum levels of visfatin and vaspin in obese children (r = 0.412, *P* < 0.001) (Fig. 1).

## Discussion

In our study, we first determined the differences in two adipocytokines, vaspin and visfatin, between lean and obese Chinese children. Obese children in our study showed significantly elevated serum visfatin and vaspin levels compared to the levels in lean controls. Next, we further studied the correlations of serum levels of vaspin and visfatin with vascular endothelial activity. We found a significant positive correlation of vaspin and visfatin levels with inflammatory and endothelial parameters, including IL-6, TNF-α, hsCRP, VCAM-1, ICAM-1, Ang-2, and E-selectin. Our multiple regression analyses independently confirmed that both vaspin and visfatin levels were associated with SDS-BMI, Ang-2, IL-6, VCAM-1, and E-selectin. Finally, we determined the association between visfatin and vaspin levels in obese children and found that these two adipocytokines were significantly and positively correlated. The results presented here indicate that the adipocytokines visfatin and vaspin play an important role in endothelial dysfunction and vascular inflammation, but they do not affect insulin resistance.

We found that vaspin and visfatin levels were significantly elevated and positively correlated with SDS-BMI and BMI in obese children. There were some differences from the results of studies conducted with adult samples, which showed that plasma vaspin and visfatin levels were not only significantly and positively correlated with BMI but were also significantly associated with WC [10, 20]. However, among these two anthropometric indicators, we found that vaspin and visfatin correlated with SDS-BMI but not with WC in obese children. In adults, WC is considered a better indicator of subcutaneous fat distribution [21], but in children, WC is still a controversial indicator for predicting cardiovascular risk because of the underlying relationship between WC and height.

Previous studies reported that vaspin and visfatin levels decrease concurrently with progression through puberty [22, 23]. However, we did not detect significant differences in vaspin and visfatin serum levels between prepubertal and pubertal children. The reason could be due to the narrow age range of the subjects and the small sample size of pubertal children in our study.

Various studies have investigated the relationship between levels of visfatin and insulin resistance in adults but with different results. Blüher indicated that plasma visfatin is related to insulin resistance, as assessed by HOMA-IR [24]. In contrast, Smith et al. and Varma et al. suggested that visfatin levels are not associated with insulin resistance [25, 26]. Another study reported that visfatin directly binds and activates the insulin receptor to induce the phosphorylation of signal transduction proteins [13]. Therefore, based on our findings presented here, we propose that visfatin exerts insulin-mimetic effects and thus enhances glucose uptake, improving insulin resistance. However, the physiological role of visfatin in the development of insulin resistance remains controversial. In our study, we did not detect an association between visfatin and FPG, 2-h PG, insulin, 2-h insulin, or insulin resistance index. This discrepancy may be explained by the fact that high levels of insulin are usually observed in adults but not in children; hence, we did not find a relationship of vaspin and visfatin with more advanced disease conditions.

One of the main goals of this study was to evaluate the potential correlation of visfatin and vaspin with early obesity-related vascular alterations. Using multiple regression analyses, we confirmed that visfatin was an independent predictor of TNF-α and IL-6, in addition to SDS-BMI and WC. It is well known that obesity is a state of low-grade chronic inflammation. A low-grade inflammatory response is considered to be the first sign of the progression of obesity-related diseases, specifically vascular alterations [27, 28]. Our results suggest that visfatin is involved in the pathological process of inflammation. Recent studies also suggested that soluble adhesion molecules, including ICAM-1, E-selectin, VCAM-1, and P-selectin, play an important role in the pathogenesis of atherosclerosis [29, 30]. In previous studies, ICAM-1 and E-selectin were used as indicators of vascular endothelial activation in obese children [31]. From our multiple regression analyses, we determined the association of visfatin with ICAM-1, E-selectin, and Ang-2. Reports state that visfatin promotes the adhesion of monocytes to endothelial cells by upregulating their expression of adhesion molecules [32]. This report confirms the role of visfatin in the development of vascular endothelial dysfunction [33]. In contrast, we did not detect a correlation between plasma visfatin levels and soluble VCAM-1. Whether circulating VCAM-1 levels are elevated in obese children, and whether this elevation has clinical significance for vascular injury, remains controversial. There has been no research conducted to understand the relationship between visfatin and Ang-2 in obese children. However, studies have confirmed that angiotensin plays an important and unique role in angiogenesis and vascular inflammation. Long-term elevation of circulating Ang-2 levels results in persistent micro-inflammation of the vascular endothelium [34].

Interestingly, it has been reported that adipocytokines that are secreted by adipose tissue stimulate vascular monocyte adhesion by activating ICAM-1 and E-selectin expression in endothelial cells [35]. In addition, many studies have discussed the link between vaspin and inflammation and atherosclerosis; however, the mechanism remains unclear [36, 37]. We observed an independent correlation between vaspin and increased hsCRP and IL-6 levels. Vaspin was correlated with hsCRP and IL-6 in obese children, indicating that these factors are involved in the systemic inflammatory response. However, published data reported a negative correlation between vaspin and CRP, demonstrating that vaspin has anti-inflammatory and anti-atherosclerotic effects in the contexts of obesity-related inflammation and CVD [38, 39]. Therefore, the relationship between vaspin and inflammatory factors remains controversial and requires further investigation.

Not all adipokines are inflammatory factors that negatively affect the progression of obesity-related diseases. Some may play a protective role in the pathogenesis of CVD. The best example is adiponectin, which can exert substantial antiatherogenic actions [40]. However, we did not find a correlation between visfatin, vaspin, and adiponectin, which is in contrast to another study that demonstrated a significant negative association among these factors. In that report, multiple regression analysis used visfatin or vaspin as the dependent parameters and did not detect any significant correlation with adiponectin. Hence, we conclude that there is no relationship between the levels of these three adipokines. We speculate that the correlation between adiponectin and visfatin and vaspin reported in the literature may not be robust. However, we found a significant inter-association of vaspin and visfatin with inflammatory and vascular endothelial function parameters. This finding suggests that visfatin and vaspin are interrelated adipocytokines and play a role in both vascular endothelial function and inflammatory cell infiltration.

This study does have a limitation that should be noted. Specifically, our study included a small sample size, which prevents the generalization of our findings. However, this sample size was sufficient to verify a significant inter-association of vaspin and visfatin with inflammatory and endothelial parameters. We recommend that large-scale studies be conducted in the future to gain more insight into the role of adipocytokines in vascular endothelial dysfunction.

## Conclusion

In conclusion, serum visfatin and vaspin levels in obese children are significantly higher than the levels in lean children. Visfatin and vaspin were significantly inter-related and associated with inflammatory and endothelial parameters. Thus, we conclude that both visfatin and vaspin play a role in vascular endothelial dysfunction and inflammatory infiltration.

## Supplementary informations

**Additional file 1: Table S1.** Clinical and biochemical features of male and female children.

**Additional file 2: Table S2.** Clinical and biochemical features of prepubertal and pubertal children

**Additional file 3: Table S3.** Anthropometric, metabolic, endothelial, and inflammatory parameters in obese children with IR and without IR

## Data Availability

The datasets used or analysed during the current study are available from the corresponding author upon reasonable request.

## Abbreviations

ABSI: A body shape index
Ang-2: Angiopoietin-2
BMI: Body mass index
DBP: Diastolic blood pressure
FPG: Fasting plasma glucose
HDL-C: High-density lipoprotein cholesterol
HOMA-IR: Homeostasis model of insulin resistance
hsCRP: High- sensitivity C-reactive protein
ICAM-1: Intercellular adhesion molecule-1
IMT: Intima-media thickness
LDL-C: Low-density lipoprotein
SBP: Systolic blood pressure
SDS-BMI: BMI s.d. score
SDS-DBP: DBP s.d. score
SDS-SBP: SBP s.d. score
TC: Total cholesterol
TG: Triglycerides
TNF-α: Tumour necrosis factor-α
VAI: Visceral adiposity index
VCAM-1: Vascular cell adhesion molecule-1
WC: Waist circumference
WHR: Waist-to-hip ratio
WHtR: Waist-height ratio
