# Supplementary material for: The role of the adipocytokines vaspin and visfatin in vascular endothelial function and insulin resistance in obese children
Source: BMC Endocr Disord. 2019 Nov 26;19:127. doi: 10.1186/s12902-019-0452-6 (PMC6878710; doi:10.1186/s12902-019-0452-6)
Supplement: Supplementary file 1 — Additional file 1: Table S1. Clinical and biochemical features of male and female children. [file 12902_2019_452_MOESM1_ESM.docx]

Supplementary Table 1. Clinical and biochemical features of male and female children

| Characteristic | Obese | | Lean | |
| --- | --- | --- | --- | --- |
|  | Boys(N=84) | girls (N=76) | Boys(N=48) | girls (N=32) |
| Age (y) | 11.43±2.31 | 10.92±2.87 | 10.48±3.45 | 11.35±2.21 |
| BMI (kg/m^2^) | 27.53±3.06 | 28.63 ± 4.02 | 17.11±3.24 | 18.02 ± 2.02 |
| SDS-BMI | 0.98±0.46 | 2.91±0.37 | 0.53±0.81 | 0.45±0.62 |
| SDS-SBP | 1.86±0.88 | 1.93±0.90 | 1.43±0.78 | 1.50±0.64 |
| SDS-DBP | 1.13±0.41 | 1.22±0.73 | 0.93±0.36 | 0.90±0.43 |
| FPG(mmol/L) | 4.86±0.68 | 5.21±0.60 | 4.32±0.89 | 4.16±0.68 |
| 2-h PG(mmol/L) | 6.59±1.35 | 6.83±1.27 | 5.96±1.25 | 6.15±1.05 |
| Insulin (lU/mL) | 16.4± 3.1 | 15.6 ± 5.2 | 13.24± 3.5 | 11.95 ± 2.4 |
| 2-h Insulin (lU/mL) | 78.7 ± 9.2 | 81.2 ± 10.7 | 72.9 ±8.1 | 74.2 ± 7.6 |
| HOMA-IR | 2.94(1.64, 5.03) | 3.06(2.16, 5.43) | 2.52(1.13,3.89) | 2.78(1.45, 4.21) |
| TG(mmol/L) | 1.38±0.46 | 1.47±0.54 | 1.12±0.42 | 1.08±0.38 |
| LDL-C(mmol/L) | 2.45±0.96 | 2.54±1.21 | 2.33±0.89 | 2.51±0.78 |
| Adapoctin(μg/mL) | 5.93±1.42 | 6.41±1.12 | 11.01±1.56 | 11.65±2.30 |
| Obestatin(pg/mL) | 138.63±18.46 | 132.82±26.18 | 241.75±35.26 | 229.53±31.15 |
| Vaspin (μg/mL) | 9.45±1.10 | 11.42±0.98 | 5.05±0.90 | 4.76±1.03 |
| Visifatin(μg/mL) | 70.32±15.31 | 75.26±12.46 | 34.62±10.52 | 38.26±9.65 |
| hsCRP(ng/mL) | 1413.43±182.32 | 1478.16±208.86 | 1042.36±182.36 | 1121.52±166.21 |
| IL-6 (pg/mL) | 31.53±6.84 | 33.51±6.26 | 19.35±4.10 | 17.56±4.85 |
| TNF-a(ng/mL) | 48.53±14.24 | 51.28±12.45 | 24.18±4.58 | 26.34±4.46 |
| ICAM-1(μg/mL) | 12.53 ±1.16 | 11.26±2.01 | 6.58 ±1.26 | 5.96 ±1.73 |
| VCAM-1(μg/mL) | 248.42±30.21 | 252.51±28.61 | 146.36±26.35 | 162±20.62 |
| Ang-2(pg/mL) | 119.34±17.21 | 123.57±14.26 | 80.36±11.25 | 84.62±13.21 |
| E-selectin(ng/mL) | 34.21±10.35 | 30.32±12.08 | 14.35±4.21 | 16.05±3.43 |

Data are expressed as mean ±s.d. or median (25^th^percentile, 75^th^ percentile). ^a^*P*<0.05; ^b^*P*<0.01 compared with obese.
