# Supplementary material for: The role of the adipocytokines vaspin and visfatin in vascular endothelial function and insulin resistance in obese children
Source: BMC Endocr Disord. 2019 Nov 26;19:127. doi: 10.1186/s12902-019-0452-6 (PMC6878710; doi:10.1186/s12902-019-0452-6)
Supplement: Supplementary file 2 — Additional file 2: Table S2. Clinical and biochemical features of prepubertal and pubertal children [file 12902_2019_452_MOESM2_ESM.docx]

Supplementary Table 2. Clinical and biochemical features of prepubertal and pubertal children

| Characteristic | Obese | | Lean | |
| --- | --- | --- | --- | --- |
|  | prepubertal (N=126) | Pubertal  (N=34) | Prepubertal  (N=59) | pubertal  (N=21) |
| Age (y) | 9.56±2.14 | 12.36±1.48^b^ | 9.24±2.85 | 12.54±2.31 ^b^ |
| BMI (kg/m^2^) | 23.32±2.84 | 29.56 ± 4.52^a^ | 16.24±3.21 | 18.89 ± 2.54 |
| SDS-BMI | 2.83±0.35 | 3.02±0.43^a^ | 0.47±0.67 | 0.51±0.82 |
| SDS-SBP | 1.84±0.78 | 1.96±0.92 | 1.34±0.43 | 1.52±0.28^b^ |
| SDS-DBP | 1.05±0.62 | 1.27±0.73 | 0.89±0.37 | 0.95±0.35 |
| FPG(mmol/L) | 4.82±0.65 | 5.25±0.68 | 4.30±0.81 | 4.35±0.62 |
| 2-h PG(mmol/L) | 6.71±1.41 | 6.62±1.18 | 5.82±1.32 | 6.31±1.16 |
| Insulin (lU/mL) | 15.62± 3.7 | 16.21±4.9 | 12.42 ± 2.4 | 13.52± 3.1 |
| 2-h Insulin (lU/mL) | 78.43± 9.8 | 80.3 ± 10.4 | 72.6 ± 8.4 | 75.6 ± 7.5 |
| HOMA-IR | 2.98(1.61, 4.82) | 3.35(2.51, 5.42) | 2.60(1.12,3.87) | 2.67(1.43, 4.41) |
| TG(mmol/L) | 1.40±0.44 | 1.48±0.53 | 1.15±0.34 | 1.06±0.31 |
| LDL-C(mmol/L) | 2.48±0.93 | 2.50±1.19 | 2.42±0.85 | 2.48±0.80 |
| Adapoctin(μg/mL) | 6.13±1.34 | 6.43±1.25 | 11.89±1.82 | 12.45±2.34 |
| Obestatin(pg/mL) | 225.62±30.25 | 243.35±33.51 | 132.64±19.32 | 137.32±25.31 |
| Vaspin (μg/mL) | 9.62±1.05 | 10.96±0.93 | 5.01±0.95 | 4.52±1.06 |
| Visifatin(μg/mL) | 70.23±14.68 | 75.92±11.52 | 35.64±11.23 | 37.51±9.26 |
| hsCRP(ng/mL) | 1408.43±186.45 | 1483.16±203.48 | 1031.85±179.62 | 1136.38±152.31 |
| IL-6 (pg/mL) | 30.18±3.56 | 34.62±6.05 | 18.89±3.92 | 17.76±4.45 |
| TNF-a(ng/mL) | 49.32±14.85 | 50.68±12.52 | 24.63±5.24 | 26.16±4.36 |
| ICAM-1(μg/mL) | 12.18 ±1.13 | 11.02±1.86 | 6.57 ±1.24 | 5.89 ±1.52 |
| VCAM-1(μg/mL) | 245.35±29.45 | 256.37±30.25 | 148.62±27.51 | 158.64±22.62 |
| Ang-2(pg/mL) | 118.53±17.21 | 128.83±14.34 | 81.52±11.26 | 85.04±9.65 |
| E-selectin(ng/mL) | 33.24±10.75 | 31.25±11.56 | 14.82±4.06 | 15.62±3.65 |

Data are expressed as mean ±s.d. or median (25^th^percentile, 75^th^ percentile). ^a^*P*<0.05; ^b^*P*<0.01 compared with obese.
