# Supplementary material for: The role of the adipocytokines vaspin and visfatin in vascular endothelial function and insulin resistance in obese children
Source: BMC Endocr Disord. 2019 Nov 26;19:127. doi: 10.1186/s12902-019-0452-6 (PMC6878710; doi:10.1186/s12902-019-0452-6)
Supplement: Supplementary file 3 — Additional file 3: Table S3. Anthropometric, metabolic, endothelial, and inflammatory parameters in obese children with IR and without IR [file 12902_2019_452_MOESM3_ESM.docx]

Supplementary Table 3. Anthropometric, metabolic, endothelial, and inflammatory parameters in obese children with IR and without IR

| Characteristic | Obese with IR  (N=28) | Obese without IR (N=132) |
| --- | --- | --- |
| Age (y) | 11.34±1.78 | 10.42±2.56 |
| BMI (kg/m^2^) | 28.89±3.62 | 27.12 ± 3.09^a^ |
| SDS-BMI | 3.12±0.53 | 2.96±0.35^a^ |
| SDS-SBP | 1.96±0.92 | 1.82±0.85^a^ |
| SDS-DBP | 1.31±0.65 | 1.18±0.52^b^ |
| FPG(mmol/L) | 5.31±0.45 | 5.03±0.51 |
| 2-h PG(mmol/L) | 6.78±1.40 | 6.65±1.32 |
| Insulin (lU/mL) | 20.23± 2.86 | 12.35 ± 2.35^b^ |
| 2-h Insulin (lU/mL) | 85.3 ± 10.7 | 60.32 ± 14.5^b^ |
| TG(mmol/L) | 1.42±0.52 | 1.38±0.48 |
| LDL-C(mmol/L) | 2.51±0.78 | 2.38±0.65 |
| Adapoctin(μg/mL) | 5.92±1.23 | 6.51±1.05 |
| Obestatin(pg/mL) | 131.14±20.15 | 136.24±22.15 |
| Vaspin (μg/mL) | 11.34±1.45 | 9.54±0.83 |
| Visifatin(μg/mL) | 72.61±14.35 | 74.12±13.42 |
| hsCRP(ng/mL) | 1465.65±201.52 | 1396.58±196.32 |
| IL-6 (pg/mL) | 33.25±5.61 | 31.26±4.56 |
| TNF-a(ng/mL) | 51.36±12.53 | 49.62±13.24 |
| ICAM-1(μg/mL) | 12.54. ±1.05 | 10.28±2.57 |
| VCAM-1(μg/mL)  Ang-2(pg/mL)  E-selectin(ng/mL) | 237.87±42.49  125.54±15.24  33.32±8.94 | 258.53±32.85  118.52±12.53  30.86±10.19 |

Data are expressed as mean ±s.d. or median (25^th^percentile, 75^th^ percentile). ^a^*P*<0.05; ^b^*P*<0.01 compared with obese.
